# Safety margins in older adults increase with improved control of a dynamic object

**DOI:** 10.3389/fnagi.2014.00158

**Published:** 2014-07-14

**Authors:** Christopher J. Hasson, Dagmar Sternad

**Affiliations:** ^1^Neuromotor Systems Laboratory, Department of Physical Therapy, Movement and Rehabilitation Sciences, Northeastern UniversityBoston, MA, USA; ^2^The Action Lab, Departments of Biology, Electrical and Computer Engineering, and Physics, Northeastern UniversityBoston, MA, USA

**Keywords:** aging, safety margin, human, dynamics, object manipulation, motor control, motor learning

## Abstract

Older adults face decreasing motor capabilities due to pervasive neuromuscular degradations. As a consequence, errors in movement control increase. Thus, older individuals should maintain larger safety margins than younger adults. While this has been shown for object manipulation tasks, several reports on whole-body activities, such as posture and locomotion, demonstrate age-related reductions in safety margins. This is despite increased costs for control errors, such as a fall. We posit that this paradox could be explained by the dynamic challenge presented by the body or also an external object, and that age-related reductions in safety margins are in part due to a decreased ability to control dynamics. To test this conjecture we used a virtual ball-in-cup task that had challenging dynamics, yet afforded an explicit rendering of the physics and safety margin. The hypotheses were: (1) When manipulating an object with challenging dynamics, older adults have smaller safety margins than younger adults. (2) Older adults increase their safety margins with practice. Nine young and 10 healthy older adults practiced moving the virtual ball-in-cup to a target location in exactly 2 s. The accuracy and precision of the timing error quantified skill, and the ball energy relative to an escape threshold quantified the safety margin. Compared to the young adults, older adults had increased timing errors, greater variability, and decreased safety margins. With practice, both young and older adults improved their ability to control the object with decreased timing errors and variability, and increased their safety margins. These results suggest that safety margins are related to the ability to control dynamics, and may explain why in tasks with simple dynamics older adults use adequate safety margins, but in more complex tasks, safety margins may be inadequate. Further, the results indicate that task-specific training may improve safety margins in older adults.

## Introduction

With aging comes an array of neuromuscular changes, such as weaker muscles, increased neural delays, and greater neuromuscular noise (Salthouse, 1996; Goodpaster et al., 2006), which together contribute to declines in motor function (Aoyagi and Shephard, 1992). Not only are the performance capabilities of older adults more limited than those of younger adults, the consequences of control errors are also more severe. This is particularly the case for locomotor activities, where a fall can cause life-threatening injuries (Berg et al., 1997). This combination of age-related neuromuscular degradations and greater costs of failure makes the maintenance of adequate safety margins a critical concern for older adults.

A common paradigm for studying safety margins is manipulation tasks, which often involve transporting a hand-held object. Here, the safety margin is typically defined as the difference between the grip force and the force required to prevent object slippage. Young adults are able to precisely regulate their grip force in response to varying object masses, staying just above the slip threshold (Johansson and Westling, 1984; Johansson and Cole, 1992). Young adults also modulate their grip force in response to load fluctuations during object acceleration (Flanagan and Wing, 1990, 1993). While older adults show similar patterns of adaptation, they generally produce higher grip forces compared to young adults (Cole, 1991; Cole and Beck, 1994; Gilles and Wing, 2003). This increases the safety margin against object slippage, which may be needed due to impaired control related to decrements in afferent function (Cole et al., 1999).

Although an age-related elevation in safety margins for object manipulation tasks is consistent with expectations, the opposite has been observed in whole-body activities, particularly locomotion and postural control. In locomotor tasks foot-obstacle clearance is critical. A small clearance implies a low safety margin against tripping. During level walking young and older adults maintain similar clearances (Winter et al., 1990). However, when the locomotor challenge is increased by adding different terrain features, older adults show reduced clearances. This includes when stepping over obstacles (McFadyen and Prince, 2002), stepping onto a raised platform (Begg and Sparrow, 2000), or descending stairs (Hamel et al., 2005). Older adults also show reduced safety margins in postural control. During upright standing older adults have reduced spatiotemporal margins of stability, which increases the risk of a fall (Slobounov et al., 1998; Van Wegen et al., 2002). Considering these observations, it appears paradoxical that in situations that warrant a larger safety margin due to more serious consequences of failure, e.g., falling, older adults use smaller, and not larger safety margins compared to young adults.

We posit that this paradox can be explained by the dynamic challenge presented by an activity or task. In grip force studies on age-related differences in object manipulation, the objects are usually rigid blocks with no further dynamics (Cole, 1991; Cole and Beck, 1994; Gilles and Wing, 2003). Changes in the load presented by the object are a linear function of the object's acceleration. On the other hand, in postural control and locomotion, the trajectory of the legs and center-of-mass are a complex function of inertial, ground reaction, and interaction forces between numerous body segments (Onyshko and Winter, 1980). In this case, older adults might be unable to control the dynamics of the body well enough to maintain adequate safety margins. In addition, in unpracticed tasks or novel dynamical experiences, individuals may be unable to utilize appropriate safety margins until sufficient skill is obtained. This is consistent with a report that in young adults improvements in performance on a virtual ball-in-a-cup transportation task are accompanied by increased safety margins (Hasson et al., 2012b).

We hypothesize that if safety margins depend on the ability to control object dynamics, then in a dynamically challenging task, older adults should have smaller safety margins than young adults (Hypothesis 1), but should increase their safety margins as their task performance improves with practice (Hypothesis 2). To test these hypotheses we used the task of moving a virtual ball-in-a-cup, representing a cup of coffee, to a target location (Hasson et al., 2012a,b; Sternad et al., 2014; Ye et al., 2014). This object has non-trivial dynamics and affords an explicit understanding of the object dynamics and a quantitative definition of the safety margin. The latter was defined as the ball energy relative to the energy needed to escape the cup. Young and older adults were asked to practice transporting the ball-and-cup to the target location in a time of exactly 2-s without letting the ball escape. This timing constraint prevented subjects from using a slowing strategy to increase safety margins but was still not fast enough to permit a range of movement strategies to achieve the task goal. Performance was assessed by the accuracy and precision of the timing error relative to the 2-s target time.

## Methods

### Participants

Nine young subjects (21–35 years) and 10 healthy older adults (65–80 years) practiced moving a virtual cup and ball to a target point in 2 s. Data on the young subjects was previously reported in Hasson et al. (2012b). The older subjects were all community dwelling, ambulatory, independent, and had no major musculoskeletal problems affecting upper body control, and no major neurological problems. Prior to participating, subjects were given a mini-mental state exam to assess cognitive function (Folstein et al., 1975); all participants scored above 23 and were therefore eligible to participate (Tombaugh and McIntyre, 1992). Before participating, subjects were informed of all experimental procedures and read and signed an informed consent document approved by the Institutional Review Board at Northeastern University.

### Ball and cup simulation

**Figure 1:**
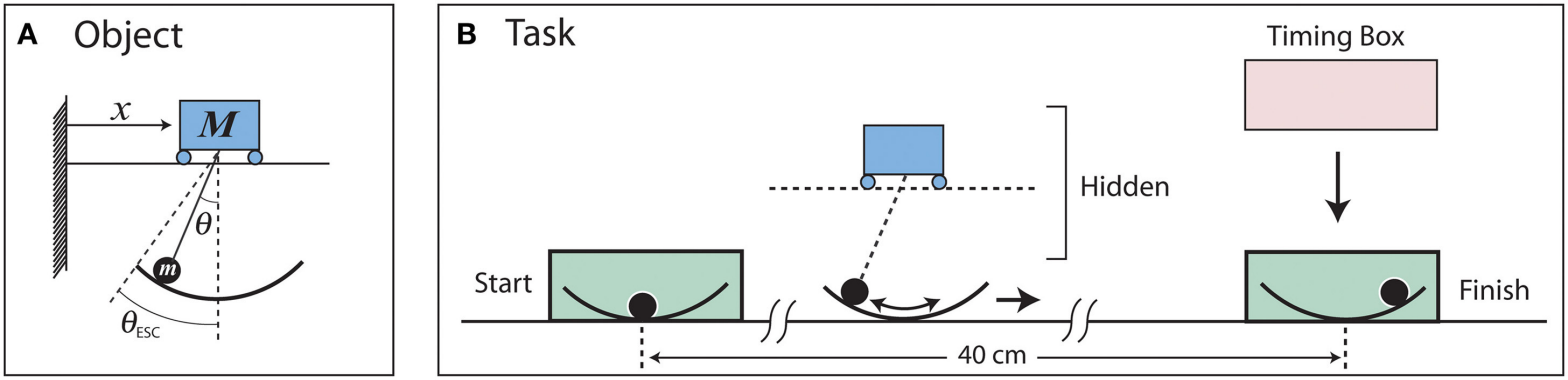
**(A)** Model of the cart and pendulum system with variables and parameters. **(B)** Task implementation. The visual display showed the pendulum bob and an arc drawn for the cup; the cart and pendulum were not shown. The cart and pendulum were haptically rendered with a robotic manipulandum. Subjects applied forces to the manipulandum, which in turn accelerated the cart (cup) and pendulum bob (ball).

The dynamics of the ball and cup object were based on that of a cart and pendulum system (Figure 1A). The cart and pendulum were haptically rendered with a robotic manipulandum (Haptic Master, Moog, Netherlands; Van der Linde and Lammertse, 2003). Details of the instrumentation are in Hasson et al. (2012b). A visual display on a rear-projection screen 2.4 m away showed the pendulum bob and a shallow “cup” drawn as an arc; the cart and the pendulum rod were not shown on the screen (Figure 1B). For subjects it appeared as if they were controlling a cup with a ball rolling inside, where the cup imposed an angular constraint on the ball. If the angle of the ball θ exceeded the maximum angle subtended by the cup, termed the escape angle θ*_ESC_ (θ_ESC_* = 35°), the ball escaped and visually “fell” out of the cup.

Subjects could manipulate the ball and cup object by applying horizontal forces to the manipulandum, which in turn accelerated the cart (cup) and the pendulum bob (ball). Forces could only be applied to the cup, and the moving ball exerted forces on the cup that subjects could feel through the manipulandum. The motion of the cup was confined to a horizontal line, and the ball could pivot about the pendulum axis of rotation, thus the system had two mechanical degrees of freedom. The cart and pendulum system behavior was governed by two equations of motion:

(1) $$\left(m+M\right)\ddot{x}=F_{A}+F_{B}$$

and

(2) $$\ddot{\theta }=\frac{\ddot{x}}{\ell }\cos\theta -\frac{g}{\ell }\sin\theta$$

where *M* is the cup mass (*M* = 3.5 kg), *m* is the ball mass (*m* = 0.3 kg), θ, $\dot{\theta }$, and $\ddot{\theta }$ are the ball angle, angular velocity, and angular acceleration, respectively; $\ddot{x}$ is the cup's horizontal acceleration, ℓ is the pendulum length (ℓ = 0.35 m), *g* is gravitational acceleration (9.81 m/s^2^), *F_A_* is an external horizontal force applied to the cup by a human actor, and *F_B_* is the horizontal reaction force of the ball on the cup, given by

(3) $$F_{B}=m\ell \ddot{\theta }\cos\theta -m\ell {\dot{\theta }}^{2}\sin\theta .$$

Details of the derivation of the equations of motion are in Hasson et al. (2012b).

### Task

Subjects were asked to transport the ball-and-cup to a spatial target located 0.4 m away in a target time of 2.0 s without letting the ball escape from the cup (Figure 1B). The 2.0 s movement time was a “comfortable” time for healthy young subjects. Because the task goal was not a limit performance, i.e., as fast as possible, a number of movement strategies could be used to complete the task in the target time; some strategies would be riskier than others, i.e., some would carry a greater risk of losing the ball. To prevent participants from spending a disproportional amount of time trying to keep the cup still in the goal region, the goal box was made “sticky” by applying a damping force *F*_D_ = −26$\dot{x}$ to the cup when both edges of the cup were inside the goal.

### Visual feedback

Two filled green rectangles were displayed, one serving as the start box and one as the goal box. The timing error was signaled with a “timing box” that descended onto the spatial target with a constant velocity so that it passed through the target at a time of 2.0 s. The timing box stopped moving when the cup was brought to a stop ($\dot{x}$ < 0.02 m/s). If the cup stopped too early (<2.0 s), the timing box was above the spatial target, if too late (>2.0 s), the timing box stopped below the spatial target. At the end of the trial, subjects were shown their temporal error in numeric form.

### Protocol

Data were collected while the participants practiced the transportation task in four blocks of 60 trials (240 total), with brief breaks between blocks.

### Energy margin

The “safety” or “riskiness” of a movement strategy was determined by computing the energy margin *EM* as

(4) $$EM=\left(E_{ESC}-TE_{BALL}\right)/E_{ESC}$$

where

(5) $$E_{ESC}=mg\ell \left(1-\cos\theta_{ESC}\right)-m\left|\ddot{x}\right|\ell \sin\theta_{ESC}+m\left|\ddot{x}\right|\ell$$

and

(6) $$TE_{BALL}=\frac{1}{2}m{\left(\ell \dot{\theta }\right)}^{2}+mg\ell \left(1-\cos\theta \right)+PSE_{BALL}$$

where

(7) $$PSE_{BALL}=\{\begin{array}{cc} \ddot{x}\geq 0 & \;-m\ddot{x}\ell \sin\theta +m\ddot{x}\ell \\ \ddot{x}<0 & \;-m\ddot{x}\ell \sin\theta -m\ddot{x}\ell \end{array}.$$

The escape energy *E_ESC_* defined the instantaneous energy threshold for ball escape. If the ball's total energy *TE_BALL_* was below *E_ESC_*, then the ball just oscillated within the cup and did not escape (assuming constant $\ddot{x}$). Otherwise, the ball would escape in the future (unless $\ddot{x}$ was changed). Note that *E_ESC_* depended on $\ddot{x}$ and therefore changed during cup transportation.

The energy margin *EM* represented how close the current ball energy was to exceeding *E_ESC_*. If *EM* was between 0 and 1, the ball did not escape. However, it would escape if *EM* was negative, assuming $\ddot{x}$ was not changed. It should be emphasized that *EM* extrapolated, i.e., it took the instantaneous energy of the ball and predicted whether the ball would escape in the future with constant $\ddot{x}$. Accordingly, *E_ESC_* was not a “hard” constraint and could be exceeded for brief periods, provided an appropriate and timely correction was made before the ball reached the cup rim.

### Dependent variables

Manipulation ability was quantified with measures related to the goal of the task: the average and standard deviation of the absolute movement timing error across trials (*MTE_AVG_* and *MTE_STD_*, respectively). The timing error was the absolute value of the difference between subjects' movement time and the 2.0 s target time. A subject with high manipulation ability should be able to reach the goal accurately (small *MTE_AVG_*) and reliability (small *MTE_STD_*). The safety margin was quantified via the average energy margin *EM_AVG_* and standard deviation of *EM* across multiple trials *EM_STD_*. All measures (*MTE_AVG_*, *MTE_STD_*, *EM_AVG_*, and *EM_STD_*) were computed across the first 30 trials (excluding the first two trials) of Block 1 and the last 30 trials of Blocks 2–4.

### Data analysis

All data analysis was performed with MATLAB (R2012b, MathWorks, Natick, MA, USA). The raw data included time histories for *x*, $\dot{x}$, $\ddot{x}$, θ, $\dot{\theta }$, $\ddot{\theta }$ and *F_A_*, which were filtered with a dual-pass fourth-order low-pass Butterworth digital filter, and used to calculate the dependent variables (*MTE_AVG_*, *MTE_STD_*, *EM_AVG_*, and *EM_STD_*). Only trials in which the target location was reached and the ball was not dropped were analyzed. To facilitate averaging movement patterns across subjects for graphical presentation, time histories were normalized to a unitary movement time (0–100%) using linear interpolation.

### Statistics

All statistical tests were performed with SPSS (Version 21, IBM Corporation, Armonk, NY, USA). A repeated-measures ANOVA was performed for each dependent variable with age as a between-subjects factor and practice block as a within-subjects factor. For all statistical tests, two subjects in the young group were identified as outliers and excluded. In contrast to the other seven subjects in the group, these subjects increased the variability of their movement patterns and used a different high-acceleration movement strategy (see Hasson et al., 2012b for more details). One subject in the older group was excluded due to excessively poor performance on the task. When appropriate, *post-hoc* comparisons were performed using Tukey's honestly significant difference test. Significance was set at *p* < 0.05 for all tests.

## Results

### General task characteristics

**Figure 2:**
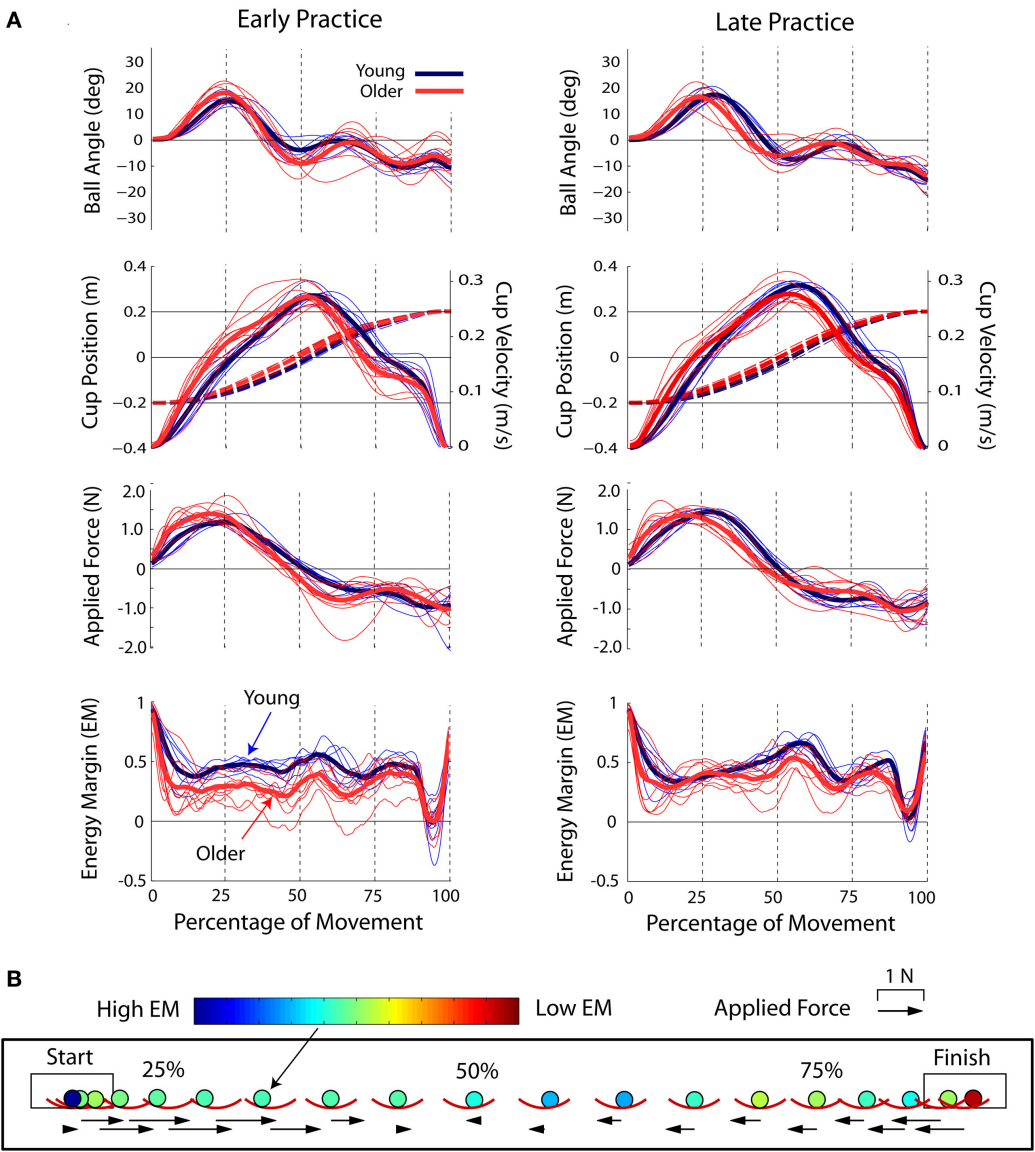
**(A)** Ball angle, cup position (dashed lines), and velocity, applied force, and energy margin for the young (blue) and older (red) subjects. Individual subjects are shown as thin lines, group averages are the thick lines. **(B)** Example of a representative ball and cup transit of an older subject in late practice [shown in **(A)**]. Images are at 5% increments of the total movement time. The color of the ball represents the energy margin; dark blue represents a high energy margin (“safe”) and dark red represents a low energy margin (“unsafe”). The arrows scale with the force applied to the cup. The size of the ball was enlarged for clarity.

The averaged ball and cup kinematics and kinetics for the young and older subjects in early and late practice are shown in Figure 2A. The ball and cup transit was divided into three phases. (1) Subjects applied a positive force to the cup, which accelerated the cup toward the spatial target and caused the ball to move backwards toward the rim of the cup (Figure 2B). These events caused the energy margin *EM* to rapidly decrease. If the force that accelerated the cup was too high, the *EM* could become negative and the ball may exceed the escape angle. (2) Subjects reduced their applied force as the cup reached the center of the workspace and the ball descended toward the center of the cup. (3) Subjects applied forces counter to the cup motion, which brought the cup to a stop at the spatial target; this was by far the riskiest part of the movement, highlighted by the rapid *EM* decrease. In early practice, the older adults applied more force to the ball at the start of each trial, causing the cup velocity to increase faster, the ball to move more, and consequently, the *EM* to decrease faster. In early practice, the ball oscillation amplitudes remained larger in the older adults until the last quarter of the movement. In contrast, in late practice, the older adults reduced these amplitudes to be similar to the young adults, and therefore the *EM* was raised.

### Failures

**Figure 3:**
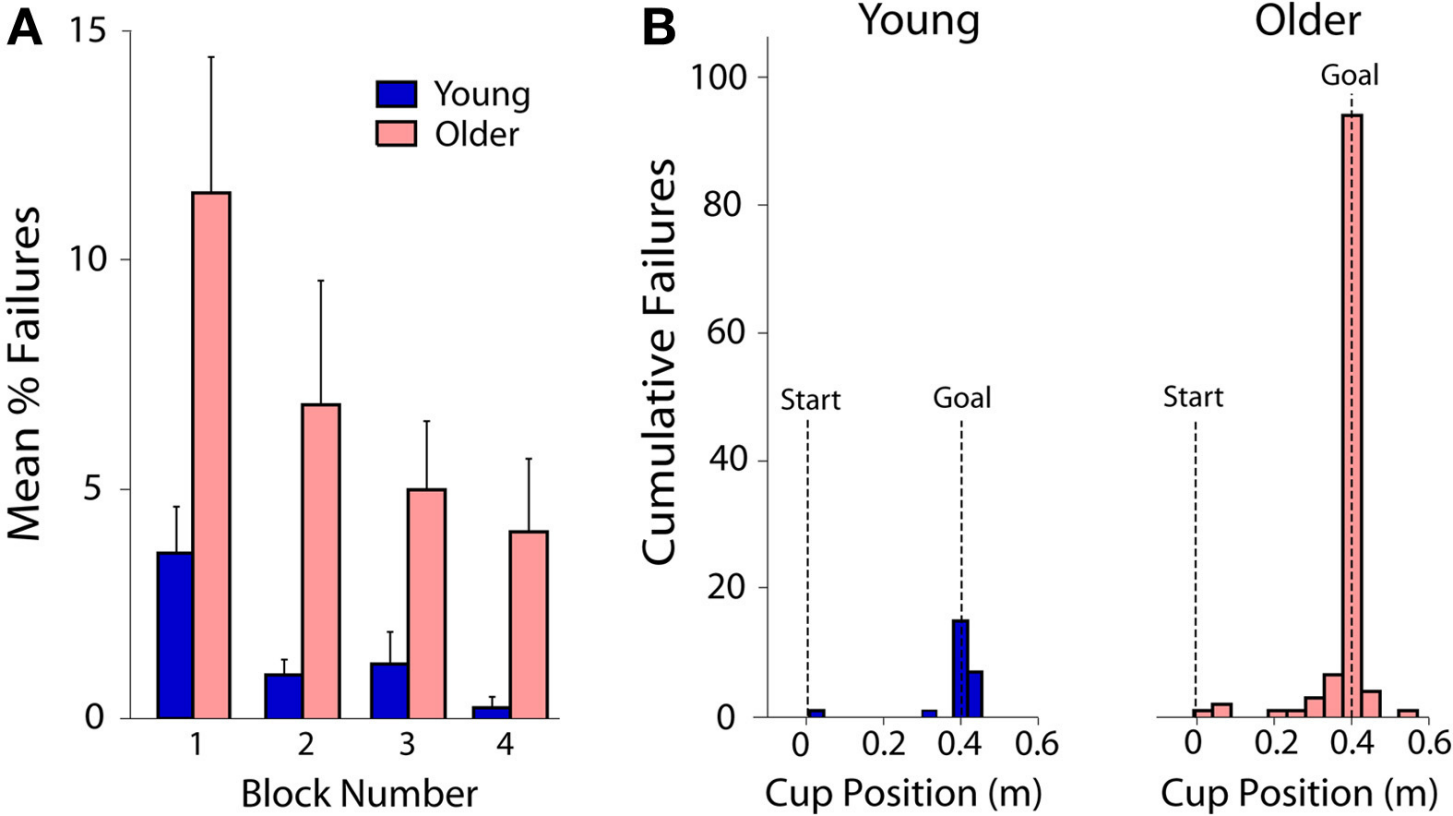
**(A)** Both young and older subjects decreased their failure rate with practice, but older adults failed more often overall. Failure was defined when the ball exceeded the escape angle. The bars show the mean percentage of failures for young (blue bars) and older (red bars) across the four blocks of practice. Error bars show standard error. Each block includes 60 trials. **(B)** Failure rate as a function of cup position for young and old subjects separately. By far the riskiest part of the ball and cup transit was stopping in the goal (at 0.4 m). Shown is the number of failed trials across all subjects as a function of the cup position at the time of failure.

Overall the older adults had a higher failure rate, i.e., lost the ball more frequently, compared to young adults [main effect *F*_(1, 15)_ = 6.8; *p* < 0.021; Figure 3A]. Both groups decreased their failure rate with practice [main effect *F*_(3, 45)_ = 5.7; *p* < 0.007], but this decrease did not differ between the groups (i.e., no interaction; *p* = 0.392). For both young and old subjects, the point at which the ball was most likely to escape from the cup was when arriving at the goal (Figure 3B).

### Task performance

**Figure 4:**
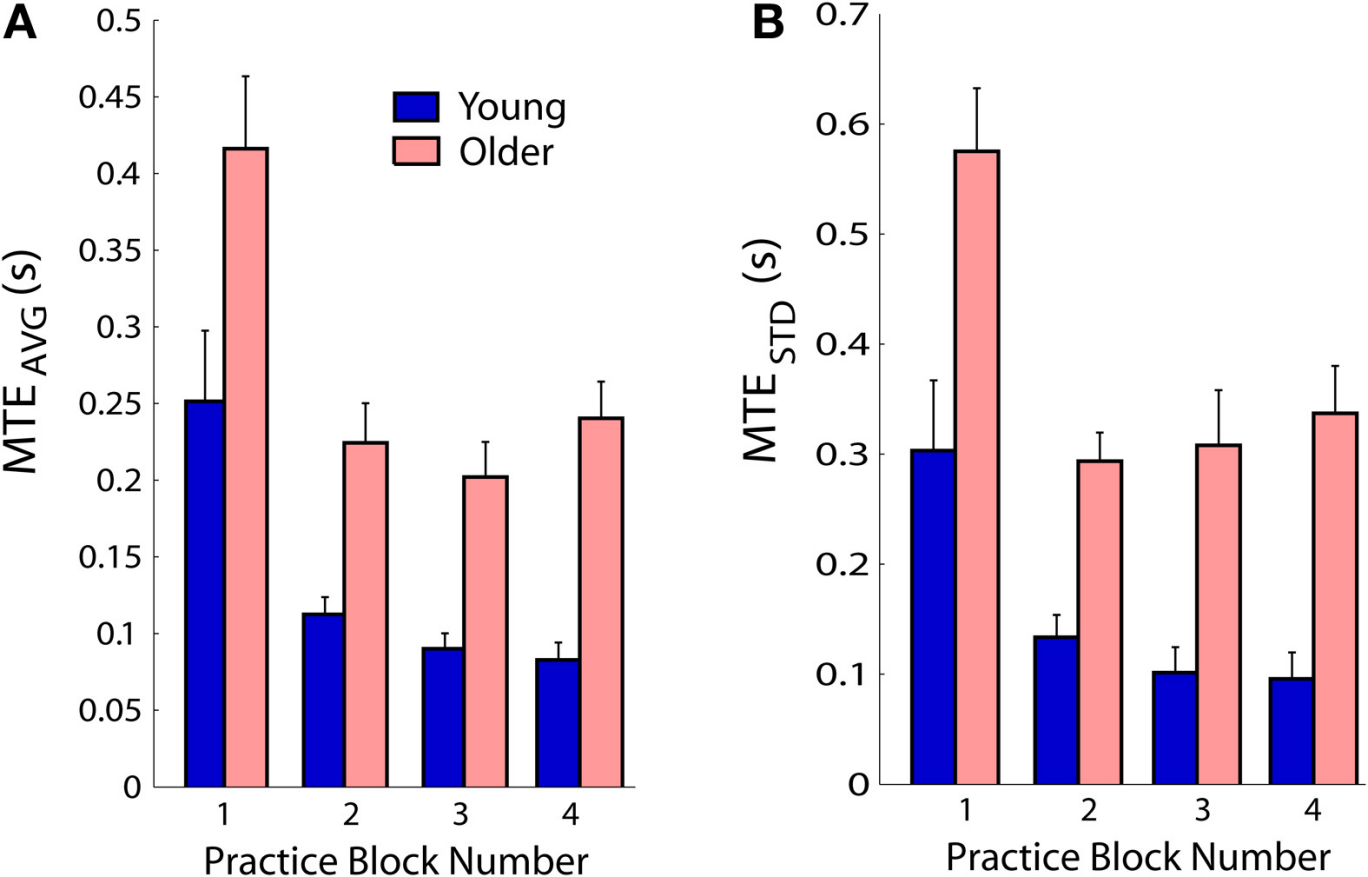
**(A)** Average movement time error *MTE_AVG_* improved with practice in both young and older subjects. **(B)** Trial-to-trial variability of movement time error *MTE_STD_* decreased. Age-related differences in manipulation ability were reflected by main effects of age for *MTE_AVG_* and *MTE_STD_*. Error bars show standard errors; each block included 60 trials.

Performance was quantified with measures of timing accuracy and precision. Both young and older subjects decreased their average timing error *MTE_AVG_* with practice [main effect *F*_(3, 45)_ = 20.2; *p* < 0.001; Figure 4A]. *MTE_AVG_* in block 1 was higher than in blocks 2–4 (*p* = 0.001), errors in block 2 were higher than block 3 (*p* = 0.034), but not different from block 4 (*p* = 0.546). Blocks 3 and 4 were not different (*p* = 0.195). The young subjects had smaller *MTE_AVG_* compared to older subjects [main effect *F*_(1, 15)_ = 29.9; *p* < 0.001]. There was no *MTE_AVG_* interaction between the two factors practice and age (*p* = 0.662). The trial-to-trial variability of the timing error *MTE_STD_* decreased with practice [main effect *F*_(3, 45)_ = 15.7; *p* < 0.001; Figure 4B]. *MTE_STD_* in block 1 was higher than in blocks 2–4 (*p* < 0.001), but blocks 2–4 were not different from each other (*p* > 0.759 for all comparisons). The young subjects had smaller *MTE_STD_* compared to older subjects [main effect *F*_(1, 15)_ = 37.5; *p* < 0.001]. There was no *MTE_STD_* interaction between the factors practice and age (*p* = 0.558).

### Energy margin

**Figure 5:**
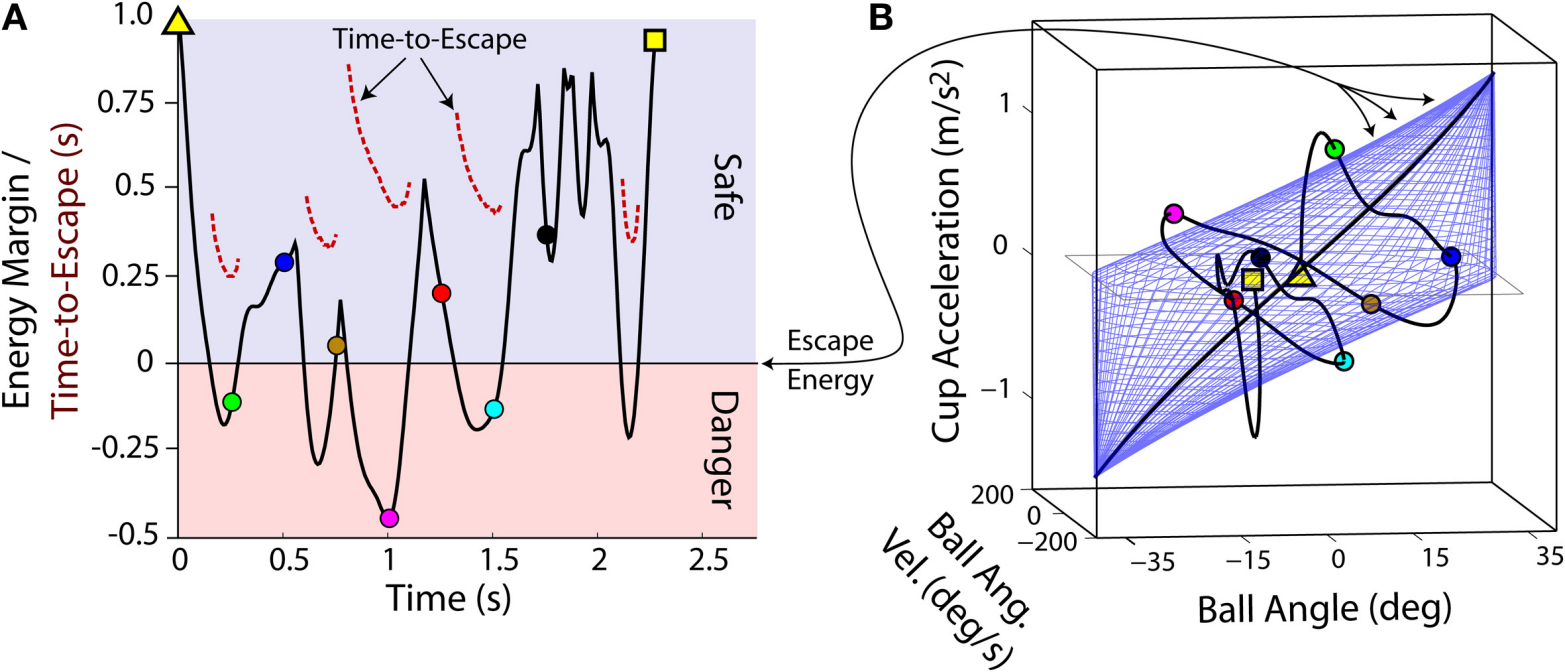
**(A)** The energy margin *EM* as a function of time (black line) in one early practice trial for one older subject. If *EM* > 0, the ball will never escape from the cup, if the current cup acceleration is maintained. However, if *EM* ≤ 0 the ball will escape from the cup, unless the cup acceleration is changed. For the latter case, the time-to-escape is shown (dashed red lines); for *EM* > 0 the time-to-escape is infinite (the ball will oscillate within the cup). **(B)** For the same trial, the three variables that determine *EM*, ball angle and angular velocity and cup acceleration, are shown in a three dimensional task execution space. The trial starts in the center (yellow triangle) and moves through the execution space as the trial progresses until the cup is stopped at the spatial target (yellow square). The blue mesh represents the critical *EM* level, i.e., where *EM* = 0. The colored circles provide timing landmarks every 0.25 s.

The safety margin was quantified in terms of an energy margin *EM*. An illustration of the *EM* and an exemplar early-practice *EM* time-history is shown for one older participant (Figure 5). The *EM* depends on the ball angle and angular velocity and cup acceleration, and therefore varied as these variables changed during a cup transit. When *EM* > 0 (light blue shading; Figure 5A), the ball will never escape from the cup, given the current cup acceleration. At the next instant in time the cup acceleration could change, which would update *EM*. An *EM* ≤ 0 (light red shading; Figure 5A) signals that the ball will escape from the cup given the current cup acceleration. Therefore a corrective action is needed to keep the ball in the cup. For the latter case, the time-to-escape is shown (dashed red lines; Figure 5A). For *EM* > 0 the time-to-escape is infinite (the ball will just oscillate within the cup).

**Figure 6:**
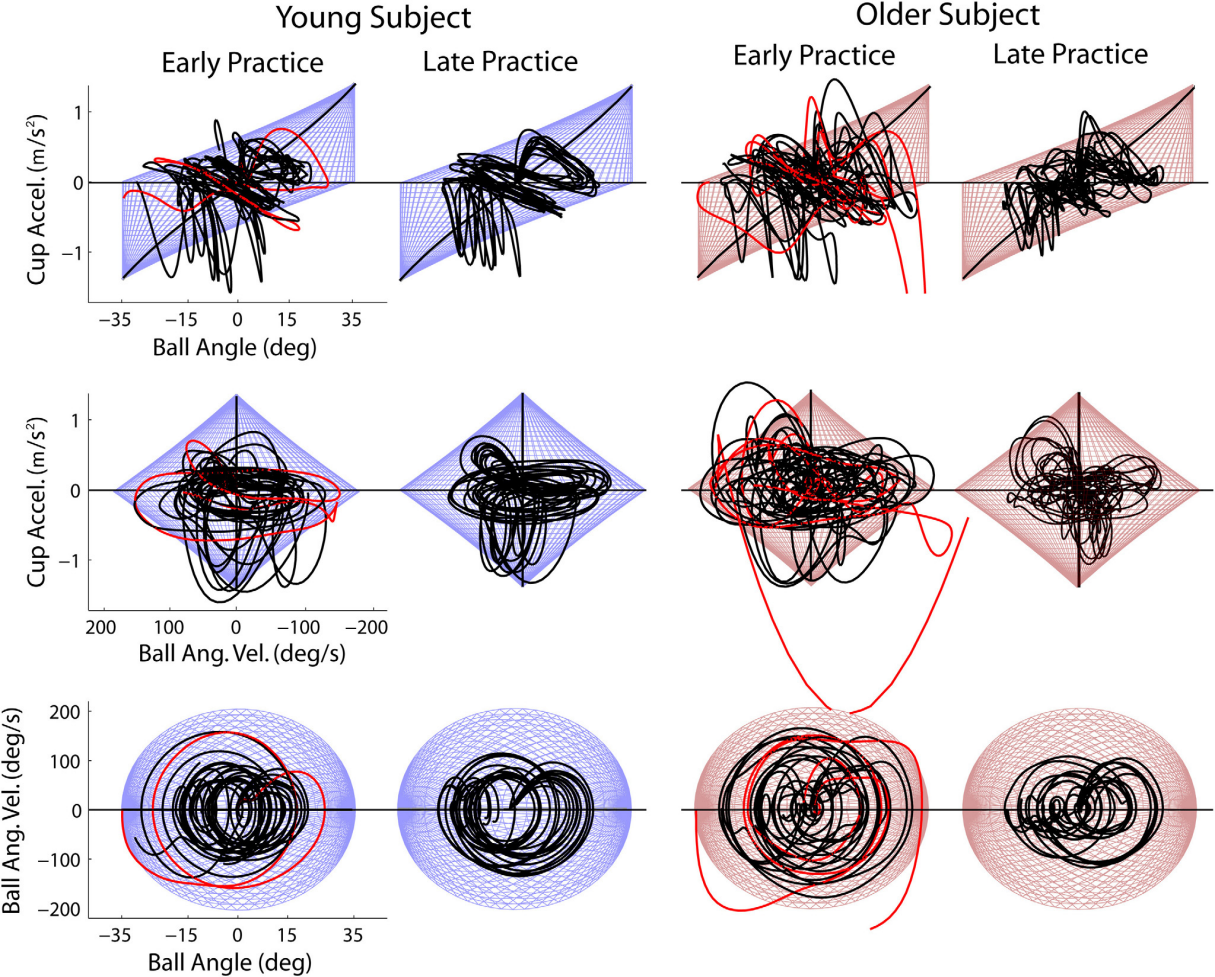
**Examples of early and late practice trials for a young and older subject**. Trajectories are plotted in the execution space, defined by ball angle and velocity and cup acceleration. Three different views of the three-dimensional execution space are shown (three rows). Trials in which the ball escaped are shown in red.

The critical energy threshold at *EM* = 0 is the escape energy *E_ESC_*. This threshold can be visualized as a two-dimensional manifold in the three-dimensional task execution space (blue mesh; Figure 5B). Each cup transit forms a trajectory in this space; as long as the trajectory stays inside *E_ESC_* manifold, the ball is not in danger of escaping from the cup. As shown in Figure 6, early in practice subjects typically had high trial-to-trial variability and frequently exceeded *E_ESC_*. However, with practice the trajectories conformed to stay largely within the *E_ESC_* manifold, except for the period of high deceleration at the end of the cup transit.

**Figure 7:**
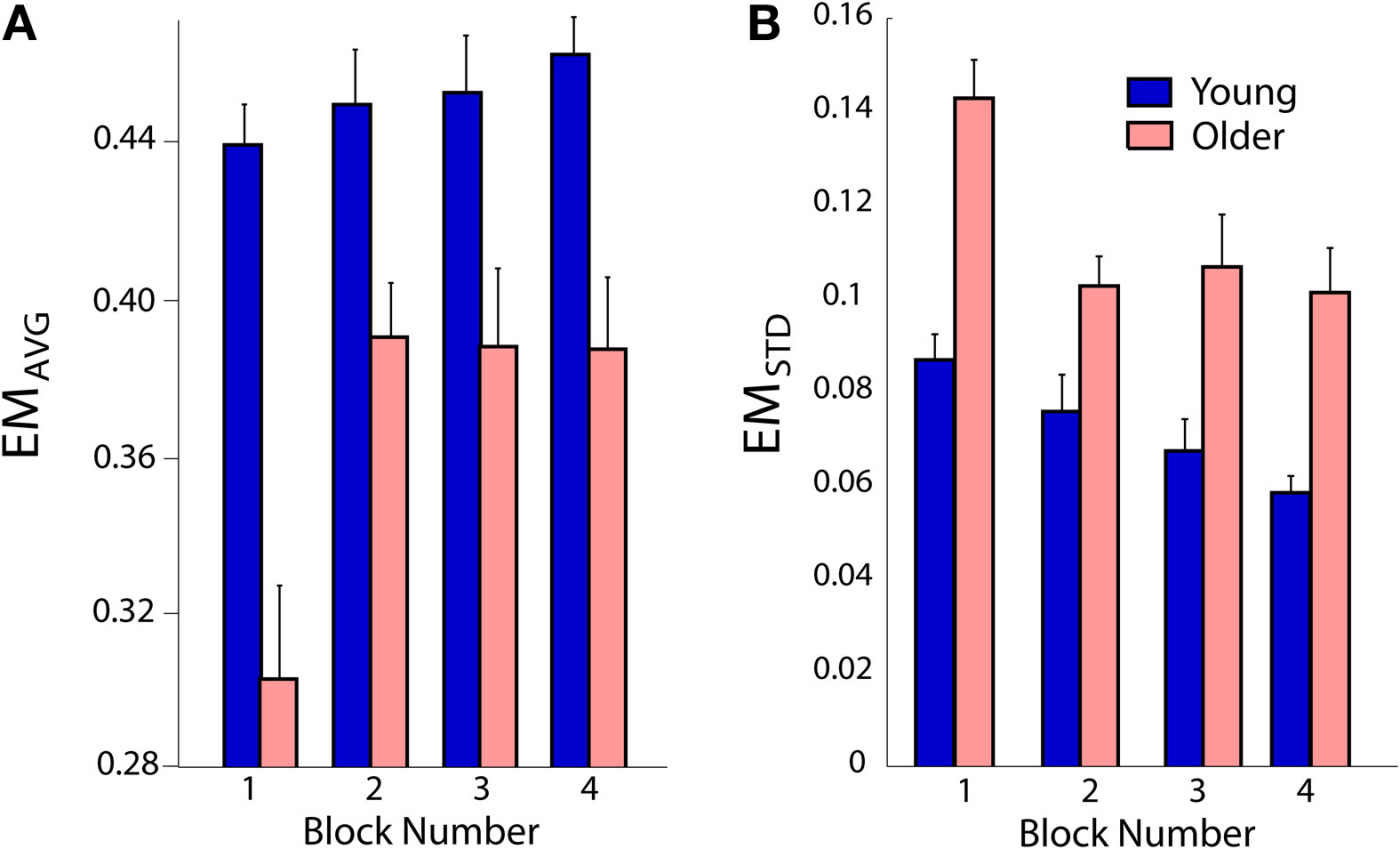
**(A)** Both young and old subjects increased their mean energy margin *EM_AVG_*. **(B)** Variability of *EM_STD_* decreased from trial to trial. Overall the older subjects had smaller and more variable energy margins. Error bars show standard error; each block included 60 trials.

*EM_AVG_* was higher in young subjects compared to older subjects [main effect *F*_(1, 15)_ = 17.6; *p* < 0.001; Figure 7A]. There was a significant effect of practice [main effect *F*_(3, 45)_ = 7.0; *p* = 0.001], but no interaction between age and practice (*p* = 0.078). For both the young and older subject groups, *EM_AVG_* in blocks 2–4 was greater than in block 1 (*p* < 0.018), but blocks 2–4 were not different from each other (*p* > 0.7 for all comparisons). *EM_STD_* was lower in young subjects compared to older subjects [main effect *F*_(1, 15)_ = 18.3; *p* < 0.001; Figure 7B], there was a significant effect of practice [main effect *F*_(1, 15)_ = 18.5; *p* ≤ 0.001], but no interaction between age and practice (*p* = 0.146).

## Discussion

Throughout practice of the virtual ball-and-cup transportation task the older adults employed a smaller safety margin, quantified in terms of an energy margin, than the younger adults, supporting hypothesis 1. Both the young and older adults increased their safety margins with practice, supporting hypothesis 2. This suggests that safety margins are learning-dependent, i.e., they were small when performance was low in early skill acquisition, but increased in parallel to other performance measures.

### Safety margins and age

The older adults used a movement strategy that was less safe than the young adults, placing them at a greater risk of failure, as shown by the greater number of ball escapes. This age-related reduction in safety margins is at odds with grip force studies that showed increased safety margins in older adults (Cole, 1991; Cole and Beck, 1994; Gilles and Wing, 2003). This could be explained by the dynamic challenges presented by the tasks. In the grip force studies (using older adults) the objects had rigid-body dynamics, i.e., the load was a linear function of the object's acceleration; the safety margins depended on only the ratio between the grip and load forces (Flanagan and Wing, 1990). In this case, the older adults may have been better able to predict the consequences of their manipulative actions, and therefore were able to maintain large safety margins. However, the increased dynamic challenge presented by the ball-and-cup object may have made it more difficult for the older adults to predict how the object would behave, reducing their ability to control the object and consequently maintain a sufficient safety margin. Note that the safety margin in the ball-and-cup task is a dynamic quantity that is a nonlinear function of three variables: the ball angle, the ball angular velocity, and the cup acceleration (Hasson et al., 2012b). The relatively smaller safety margins in the older adults is consistent with observations from whole-body tasks, such as posture and locomotion, in which older adults show reduced safety margins compared to younger adults (Slobounov et al., 1998; Begg and Sparrow, 2000; McFadyen and Prince, 2002; Van Wegen et al., 2002; Hamel et al., 2005). Together, the results of these studies suggest a link between the ability to control the dynamics of an object and the use of safety margins.

### Safety margins and task performance

The connection between manipulation ability and safety margins is strengthened by the changes observed throughout practice. Despite having overall lower safety margins and performance (accuracy and precision), the older adults increased their safety margins by about 30% and improved performance by about 50%. These changes occurred almost entirely within the first half of task practice. In contrast, the young subjects showed much smaller increases (approximately 7%), and continued to improve their performance and increased safety margins throughout practice. The smaller relative increase in the younger adults could be due to a ceiling effect. Increases in the safety margin were paralleled by a decrease in the task failure rate, which over practice decreased by more than half in the older subjects. In young adults, it was reduced to almost zero. While changes in older subject task performance and safety margins leveled off with practice, the failure rate continued to decrease. This could be related to the binary nature of the failure rate, a large decrease in the failure rate could arise from a very small change in the safety margin if close to the safety margin threshold. Indeed, as shown in Figure 2, both young and older subjects came very close to the threshold as they brought the cup to the goal. This remained a “dangerous” point even after the practice period. Note that the safety margin does not necessarily increase with improvements in task performance, i.e., the safety margin could vary independently from the goal (accuracy and precision of the timing error) due to task redundancy afforded by the specification of a 2-s target time.

The reduced performance and lower safety margins of the older adults may be due to a combination of physiological limitations, such as less reliable sensory information (Light, 1990), slower sensory integration and cognitive processing (Myerson et al., 1990; Bashore et al., 1997), and increased neural noise and delays (Laidlaw et al., 2000; Pannese, 2011). Together, these changes may limit the ability of older subjects to make fast compensatory actions to keep the ball from escaping the cup, especially at the end of the movement, where the older adults had the lowest safety margin and lost the ball most frequently. Although the data are consistent with the hypothesis that safety margins are related to the ability of the older adults to control dynamics, a more nuanced view would suggest that additional factors might play a role in regulating safety margins. Maintaining large safety margins may incur an energy cost, i.e., lifting the foot high over an obstacle would require more effort than just clearing the obstacle (Chou et al., 1997). Large safety margins may also limit maneuverability, e.g., in posture keeping a large stability margin reduces the ability to make quick postural changes (Huang and Ahmed, 2011).

### Implications and applications

The task constraints imposed in the experiment raise the question of how these results may relate to the real world. The results show that, given a shallow cup and constrained movement time, older adults are less safe than younger adults. Instead of increasing their safety margins by improving their ability to control the dynamics of the ball and cup object, in real life older adults could take other steps to increase their safety margins. They could choose a very deep cup, place a lid on the cup, or move more slowly. The latter could reduce the cup accelerations, leading to less ball movement and therefore a greater safety margin. Along the same lines, older adults can increase the safety margins in activities like posture and locomotion by either walking more slowly and/or increasing the size of their base of support.

Experimentally, increasing the dynamic challenge presented to the subjects by adding movement time constraints had two key benefits. First, by stressing the neuromuscular system, limitations become clearer (Guadagnoli and Lee, 2004). If we had allowed subjects to slow down, it would have been unlikely that the age differences in safety margins would have been as large. In locomotor studies, if subjects walk on a level ground with no obstacles or cognitive challenges, there are no age differences in the safety margin (Winter et al., 1990). Second, there may be some instances in daily life, which preclude options, such as “slowing down.” For example, while crossing the road one might need to quickly step up onto a curb due to errors in judging the speed of traffic. In this case, an inadequate safety margin may cause a fall leading to a serious injury or death.

## Limitations

One of the challenges associated with the analysis of dynamically complex systems is the definition of the safety margin. For the ball-and-cup task we chose to define the safety margin in terms of the ball energy relative to escape. Clearly, there are other ways that the safety margin could be defined. For example, simpler versions could be just the angular distance of the ball from the cup rim, and/or the angular velocity of the ball as it approaches the rim. Our measure considers the ball angle, angular velocity, and cup acceleration, as all three variables determine the ball energy and thereby the risk of escape. More details about our rationale are provided in Hasson et al. (2012b).

## Conclusions

This study demonstrated that in a constrained virtual ball-and-cup transportation task, older adults had less manipulation skill and utilized smaller safety margins compared to younger adults. However, with practice the older adults were able to improve their skill and increase their safety margins. These findings suggest that safety margins are related to the ability to control dynamics, and may explain why in tasks with simple dynamics older adults use adequate safety margins, but in more complex whole-body tasks safety margins are inadequate. Further, the results indicate that task-specific training may improve safety margins in older adults.

### Conflict of interest statement

The authors declare that the research was conducted in the absence of any commercial or financial relationships that could be construed as a potential conflict of interest.
